# The mitochondrial genomes of sponges provide evidence for multiple invasions by Repetitive Hairpin-forming Elements (RHE)

**DOI:** 10.1186/1471-2164-10-591

**Published:** 2009-12-09

**Authors:** Dirk Erpenbeck, Oliver Voigt, Gert Wörheide, Dennis V Lavrov

**Affiliations:** 1 Department of Earth- and Environmental Sciences, Palaeontology & Geobiology and GeoBioCenter LMU, Ludwig-Maximilians Universität München, Richard-Wagner-Str. 10, 80333 München, Germany; 2 Department of Ecology, Evolution, and Organismal Biology, Iowa State University, 343A Bessey Hall, Ames, IA 50011, USA

## Abstract

**Background:**

The mitochondrial (mt) genomes of sponges possess a variety of features, which appear to be intermediate between those of Eumetazoa and non-metazoan opisthokonts. Among these features is the presence of long intergenic regions, which are common in other eukaryotes, but generally absent in Eumetazoa. Here we analyse poriferan mitochondrial intergenic regions, paying particular attention to repetitive sequences within them. In this context we introduce the mitochondrial genome of *Ircinia strobilina *(Lamarck, 1816; Demospongiae: Dictyoceratida) and compare it with mtDNA of other sponges.

**Results:**

Mt genomes of dictyoceratid sponges are identical in gene order and content but display major differences in size and organization of intergenic regions. An even higher degree of diversity in the structure of intergenic regions was found among different orders of demosponges. One interesting observation made from such comparisons was of what appears to be recurrent invasions of sponge mitochondrial genomes by repetitive hairpin-forming elements, which cause large genome size differences even among closely related taxa. These repetitive hairpin-forming elements are structurally and compositionally divergent and display a scattered distribution throughout various groups of demosponges.

**Conclusion:**

Large intergenic regions of poriferan mt genomes are targets for insertions of repetitive hairpin- forming elements, similar to the ones found in non-metazoan opisthokonts. Such elements were likely present in some lineages early in animal mitochondrial genome evolution but were subsequently lost during the reduction of intergenic regions, which occurred in the Eumetazoa lineage after the split of Porifera. Porifera acquired their elements in several independent events. Patterns of their intra-genomic dispersal can be seen in the mt genome of *Vaceletia *sp.

## Background

Organellar genomes display a tendency of size reduction (deletional bias) [1]. This tendency manifests itself in the loss of mitochondrial (mt) protein genes or their relocation to the nucleus, and in the loss of intergenic non-coding sequences. For example, comparison between animal mt genomes and that of the choanoflagellate *Monosiga brevicollis *revealed that a major reduction of mtDNA has taken place in the animal lineage, which involved the translocation of mitochondrial genes into the nucleus and dramatic size reduction of intergenic regions (IGR) [2]. Indeed, the IGRs account for almost 50% of the 76 kb mt genome of *M. brevicollis *[3], while the poriferan genomes examined so far from Demospongiae, Homoscleromorpha and Hexactinellida (no mt genome is yet available for class Calcarea) possess less than 24% IGRs (in *Axinella corrugata *[4]). Similarly, the number of genes is reduced from 55 in *M. brevicollis *[3] to 40 - 18 in demosponges [5]. This diminution of mt DNA culminates in bilaterian animals, where IGRs are frequently absent (with adjacent genes often overlapping each other) and occasionally genes being lost. As a result, the majority of non-coding nucleotides in bilaterian mt genomes is located in a single control region, which contains important elements for the replication of mtDNA (see [6] for an overview). Although such control region has not unambiguously been identified in non-bilaterian Metazoa, some characteristic features like repetitive sequences [7], conserved sequence blocks and potential secondary structures for the initiation of replication [8] have been found in the mt genome of *Acropora tenuis *(Cnidaria [9]). In Porifera, non-coding regions with repetitive features are speculative control regions, but similarity to their eumetazoan counterparts and conservation among different species are low [10,11].

The structure and biological function of mt genes is well-studied owing to their pivotal role in respiration and oxidative phosphorylation and several of these genes are frequently used as phylogenetic markers. By contrast, elements involved in the replication and expression of mtDNA have been investigated only in a few species [8]. However, it has been observed that mtDNA outside of bilaterian animals usually contains multiple IGRs of similar length and these genomes often harbour numerous repetitive sequences [12]. Such repeats occur mostly in intergenic regions but were also found inside protein coding or ribosomal RNA genes (e.g. [13-15]). These mt repetitive sequences can comprise all classes of their nuclear counterparts, which include direct-, dispersed-, inverse-, tandem- and satellite-like repeats (see [12] for an overview). Frequently, repetitive mtDNA elements have a potential to form a particular secondary structure with stems and loops. Conserved and potentially mobile palindromic repeats are well known from non-metazoan mtDNA [16]. Most abundant are single hairpin-forming motifs (e.g. [17]), but double hairpin elements were also found [12,18]. In the following we will refer to such elements as Repetitive Hairpin-forming Elements (RHE).

Despite their regular occurrence in fungi, plants, and other eukaryotes, no adaptive function of RHEs is known, although their potential roles as control elements in mRNA processing and translation have been discussed. For example, inverted repeat sequence elements are often found in the 3' - untranslated regions of mRNAs and have been suggested as candidate structures for RNAse access [19]. Alternatively, inverted repeats forming stem-loop structures at 3' termini of mRNAs have also been found to be stabilizing signals in both bacteria and chloroplasts [20-23]. Besides their involvement in RNA processing, repeat structures and, in particular, (double-) hairpin elements could facilitate recombination and lead to mt genome reorganization. This function would be analogous to G-C rich clusters in the mt genome of *S. cerevisiae *[12], which can be folded into several different motifs of stem-and-loop structures with high similarity [24] and are regarded as preferential recombination sites [25-28]. Finally, mt repetitive elements, like their cytoplasmatic counterparts, could have simply evolved out of transposable elements or by errors during mt DNA replication [12].

These insights from non-metazoan opisthokonts indicate that mt IGRs may play an important role in the evolution of the metazoan mt genome. To our knowledge there is no information about the presence of RHE in choanoflagellates, Ichtyosporea and Placozoa. In Bilateria, the impact of RHE was reduced concurrently with the condensation of the mt genome to the highly compact circular ~16 kb DNA molecule, as present today in most animal phyla. To investigate the potential impact of RHE on early metazoan mt DNA evolution, we studied the IGRs in all available mt genomes from sponges, which form the basal divergence with Eumetazoa within the animal lineage [29]. In this paper, we will initially focus on the mt genomes of keratose demosponges (Keratosa), which, together with the Myxospongiae, form the sister group to all other extant demosponge lineages [5,30] and then extend our analysis to other groups of demosponges and sponges in general.

The keratose sponge order Dictyoceratida encompasses sponges with a high morphological diversity. Most genera, such as *Hippospongia or Ircinia *possess a purely organic skeleton of spongin fibers. Recent molecular data, however, demonstrated that *Vaceletia*, a sponge with a 'sphinctozoan' -type skeleton, i.e. with a hypercalcified (so-called "coralline") mineral skeleton of aragonite with trabecular inner structure, likewise belongs to the Dictyoceratida [5,31], a taxon that normally is devoid of biomineral-production. In this context we present the mt genome of an additional non-coralline dictyoceratid sponge, *Ircinia strobilina*, which allows us to get a better insight on intra-ordinal variation of sponge mtDNA.

## Results

### Organization of dictyoceratid demosponge mtDNA

**Figure 1:**
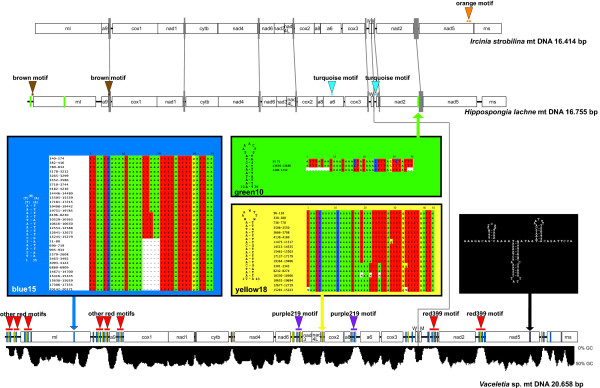
**Dictyoceratida mtDNA**. Schematic and linearized view of the three dictyoceratid mt genomes. The grey vertical bars indicate IGRs of high similarity between the taxa as connected by grey lines. Coloured horizontal bars (also highlighted with triangles and motif names) indicate repetitive regions inside the genomes. Coloured vertical bars (also highlighted with triangles and motif names) inside the genomes display putative RHEs. Their corresponding secondary structure and alignment is provided inside the boxes of the same colour. The GC-content of the *Vaceletia *mt genome is indicated by the black field below the structure. The numbers in the sequence names refer to their position in the mt genome. The numbers at the structures refer to their position in the alignments.

The mt genomes of dictyoceratid demosponges *Ircinia strobilina*, *Hippospongia lachne *and *Vaceletia *sp. comprise 16,414, 16,755, and 20,658 bp, respectively (Figure 1). They code for the standard 14 demosponge protein genes (which include *atp9*), small and large subunit ribosomal RNA (*rns *and *rnl*) but only two tRNAs (*trnW(uca) *and *trnMf(cau)*). The latter represent the minimally required suite of mt tRNA genes if all other tRNAs are imported from the cytoplasm: *trnW(uca) *is needed to accommodate for sponge (and mold and cnidarian) deviation from the universal mitochondrial code [32] while a specialized *trnMf(cau) *is necessary for the initiation of translation (see also [33]). This reduced suite of tRNA genes is identical to that found in most Cnidaria [34]. All three mt genomes display an identical gene order, including the position of tRNAs. The selection pressures on the coding genes is also very similar in these genomes as the ratio of synonymous/nonsynonymous substitutions per site is estimated as: *Vaceletia *sp. -* I. strobilina*: (0.30/0.03); *Vaceletia *sp. - *H. lachne*: (0.27/0.03); *I. strobilina *- *H. lachne*: (0.17/0.02).

### Structure of intergenic regions in dictyoceratid demosponges

Dictyoceratid intergenic regions show large length differences, resulting in mtDNA size variation of approximately 20% among the analyzed species. *Vaceletia *sp. possesses IGRs totalling 4,520 bp, compared to 1,566 bp and 871 bp in *H. lachne *and *I. strobilina*, respectively. IGRs in the latter two species display a high degree of similarity (Figure 1 grey vertical bars), which is highest at IGR termini and decreases towards their centres. In *Vaceletia *the IGRs connecting *rnl *- *atp9 *(700 bp), and *trnM(cau) *- *nad2 *(657 bp) are particularly long. There is no evidence for additional ORFs in any of these regions.

### Repetitive elements in the IGR of dictyoceratid demosponges

Several long duplications exist within the mt IGR of *Vaceletia*. Two IGR stretches of 339 bp between *nad5 *- *nad2 *and *trnMf(cau) *- *nad2 *are identical (Figure 1, red horizontal bars "*red 339 motif*"). Parts of this motif are also present in other IGRs: *rnl*-*atp9 *(108 and 219 bp), *atp9*-*cox1 *(222 bp), *rns*-*rnl *(214 and 88 bp) and even within *rnl *(236 bp) (Figure 1, red horizontal bars "*other red motif*s"). Another duplicated region of significant length (219 bp) is located in the IGRs connecting *nad6 *- *nad3 *and *atp8 *- *atp6 *(Figure 1: purple horizontal bars "*purple219 motif*").

These long repetitive regions are more than 90% AT, which is much higher than for the other regions of the mt genome (Figure 1, black curve under the *Vaceletia *mt genome). A closer analysis of the AT-rich repetitive regions reveals the presence of repetitive small, subunits, which form perfect hairpin structures. Two different RHE types have been detected: the first type consists of two uninterrupted complementary regions of 15 bp each, occasionally separated by 5 bases, which will in the following be referred to as "*blue15*" (Figure 1). We have found 32 complete, i.e. hairpin-forming copies of this type. The second type consists of two complementary regions of 18 bp with a 7 bp terminal loop (Figure 1). The *Vaceletia *mt genome contains 17 complete copies of this type, which will in the following be referred to as "*yellow18*". Besides their length, the *blue15 *and *yellow18 *complementary regions differ from each other by few complementary substitutions. In addition, several incomplete *blue15 *and *yellow18 *RHE (i.e. without complementary region in close proximity) have been detected (not shown). The *blue15 *and *yellow18 *RHE often occur in tandem resulting in double hairpin and, in some cases, multiple hairpin structure. Both hairpin motifs also occur in *rnl*, while the *blue15 *type is also present in *rns*.

Compared to its homologues in dictyoceratid species *Hippospongia lachne *and *Ircinia strobilina*, *nad5 *of *Vaceletia *sp. possesses a 90 bp AT-rich insert (Figure 1: black vertical bar) with a potential to form a triple hairpin structure (Figure 1, black box). Similar insertions have been found in *nad2 *and *nad5 *of *Axinella corrugata *(see below). Considering the fact that they do not cause frameshifts it is likely that these elements are not spliced out of the transcripts. Also, insertions in *nad5 *are not unusual in Order Dictyoceratida - *Ircinia strobilina *possesses a stretch of 16 almost identically duplicated amino acids at the 3' end (YVT(VW/GS)GIEYAEVPEYL), Figure 1, orange elements "*orange motif*"). In the *Ircinia strobilina *mt genome this is the only repetitive feature.

In contrast to our findings in *Vaceletia*, the mt genome of *Hippospongia lachne *possesses only three repetitive motifs. The first forms a RHE type with a 10 bp helix and a 7 bp loop in three copies (Figure 1: "*green10*"), of which two occur in IGRs and one in *rnl *(Figure 1: green vertical bar). In addition, a 31 bp long fragment of *atp6 *has been copied into the IGR between the tRNA genes (Figure 1, "*turquoise motifs*") and a 42 bp fragment is identical in the intergenic regions 5' of *rnl *and *cox1 *(Figure 1, "*brown motif*"). Both of the latter elements do not form hairpins.

### Repetitive and other unusual elements in mtDNA of other demosponges

#### Keratosa

**Figure 2:**
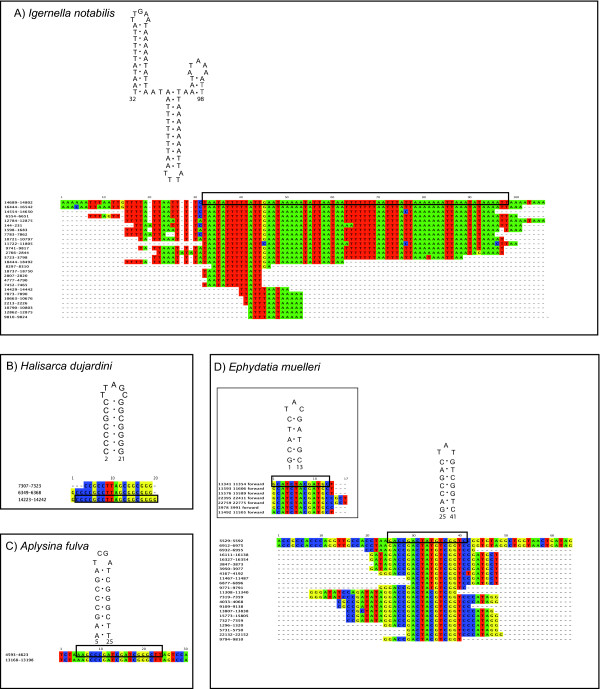
**RHEs in miscellaneous demosponges**. Secondary structures and their corresponding alignments of RHEs in A) *Igernella notabilis *(Dendroceratida), B) *Halisarca dujardini *(Halisarcida), C) *Aplysina fulva *(Verongida) and D) *Ephydatia muelleri *(Spongillina). The numbers in the sequence names refer to their position in the mt genome. The numbers at the structures refer to their position in the alignments. The black box indicates the particular fragment region for which the secondary structure is given. Alignable flanking regions are displayed.

Dendroceratida is sister order to Dictyoceratida - both form the taxon Keratosa. MtDNA of the dendroceratid *Igernella notabilis *possesses IGRs of 3,586 nucleotides. The most remarkable feature of these IGRs is the presence of a highly AT-rich RHE in 14 complete, i.e. double- or triple hairpin-forming copies (Figure 2A), besides several potentially incomplete copies. Complete RHE copies are found in all intergenic regions with the exception of *cox1-nad1, cox2-atp8, cob-nad6, nad3-nad4l, nad4-trnM, nad2-trnW(uca) *and *rns*-*atp9*, but twice between *trnW(uca) *and *nad5*, and one such element is inserted into *rnl*.

#### Myxospongiae

Sponges of the orders Halisarcida, Chondrosida and Verongida are combined into the Myxospongiae [30] and form a sister group to Keratosa in molecular phylogenies [5,30]. The IGR of *Halisarca dujardini *(Halisarcida) contains 1,328-noncoding base pairs with only one potential RHE type, 17 bp long and GC-rich (Figure 2B). The RHEs are located in the IGRs connecting *cox3 *- *trnQ(uug)*, *atp9 - trnS(gcu) *and, furthermore, inside a variable region of *nad2*. *Chondrilla nucula *(Chondrillidae) does not possess any remarkable repetitive feature in its 1,377 bp intergenic regions. *Aplysina fulva *(Verongida) has 1,478 intergenic nucleotides, with just a single RHE motif of two complementary 10 bp strands. It occurs in two copies in the IGRs connecting *trnH(gug) - nad4 *and *atp6 *- *trnR(ucu) *(Figure 2C).

#### Marine Haplosclerida

Mt genomes of marine haplosclerids *Callyspongia plicifera *(1,100 bp), *Xestospongia muta *(938 bp) *Amphimedon compressa *(887 bp) and *Amphimedon queenslandica *(2,413 bp) lack RHEs. However, *Amphimedon queenslandica *(2,413 bp) mtDNA contains a 6× tandemly repeated 12 bp motif between the two rRNA genes, a feature that remains unique among Porifera [10].

#### The "G4" clade

**Figure 3:**
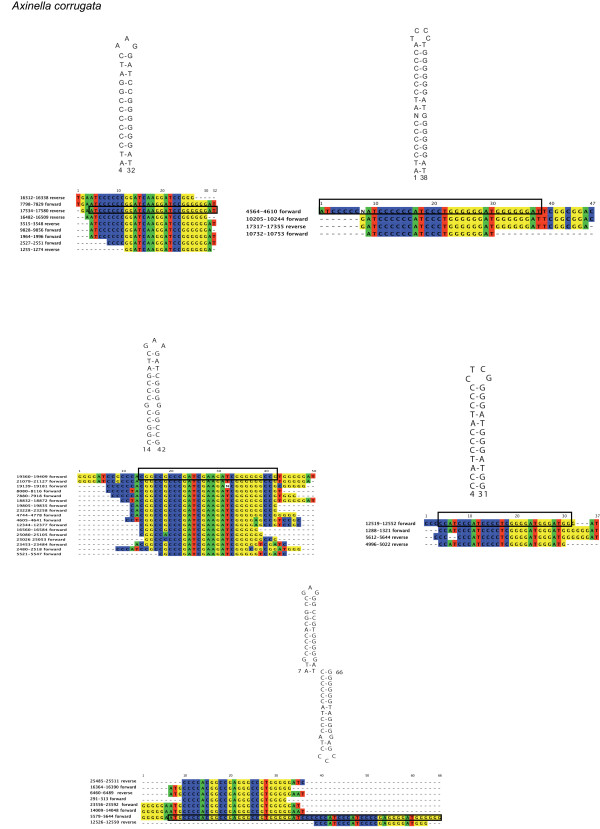
**RHEs in the demosponge *Axinella corrugata***. Secondary structures and their corresponding alignments of RHEs in *Axinella corrugata *(Halichondrida). The numbers in the sequence names refer to their position in the mt genome. The numbers at the structures refer to their position in the alignments. The black box indicates the particular fragment region for which the secondary structure is given. Alignable flanking regions are displayed.

**Figure 4:**
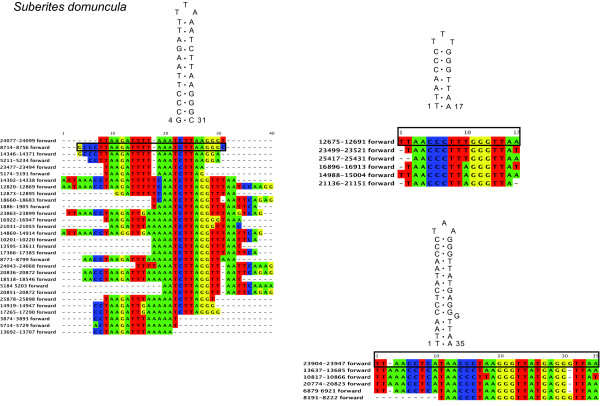
**RHEs in the demosponge *Suberites domuncula***. Secondary structures and their corresponding alignments of RHEs in *Suberites domuncula *(Hadromerida). The numbers in the sequence names refer to their position in the mt genome. The numbers at the structures refer to their position in the alignments. The black box indicates the particular fragment region for which the secondary structure is given. Alignable flanking regions are displayed.

According to molecular markers [5,30], the remaining demosponge lineages belong to the clade "G4" yet to be named and resolved. The mt genome of the freshwater sponge *Ephydatia muelleri *(Haplosclerida, Spongillina) possesses a large amount of intergenic nucleotides (5,242 bp IGR), containing RHEs with equal GC/AT- composition (Figure 2D). The most frequent RHE consists of two 8 bp complementary regions and is present 19 times in IGRs and *rnl*. Occasionally, RHEs form a triple hairpin structure. The mt genomes of *Cinachyrella kuekenthali *(490 bp IGR), *Geodia neptuni *(Astrophorida, 340 bp IGR), *Topsentia ophiraphidites *(Halichondrida, 1,856 bp IGR), *Negombata magnifica *(Poecilosclerida, 2,064 bp IGR), *Ectyoplasia ferox *(Poecilosclerida, 530 bp IGR), *Iotrochota birotulata *(Poecilosclerida, 1,355 bp IGR), *Ptilocaulis walpersi *(Halichondrida, 1,003 bp IGR), *Agelas schmidti *(Agelasida, 2,134 bp IGR) and *Tethya actinia *(Hadromerida, 1,485 bp IGR) do not possess any peculiar repetitive features according to our search criteria, except for a 91 bp long fragment of *nad1 *copied into the IGR 5' of *nad2 *of *I. birotulata*. In contrast, *Axinella corrugata *(Halichondrida, 6,077 bp IGR) possesses a large variety of GC-rich (>70%) RHEs, including double and triple hairpin motifs (see Figure 3). In several instances, these repeats are tandemly arranged into multi-repeat motifs. Repeats reach a length up to 84 bp and are present in almost all intergenic regions of *A. corrugata*, as well as inside *nad2, nad5 *and both ribosomal RNA genes. For *Suberites domuncula *(Hadromerida, 6,519 bp IGR), numerous repetitive hairpin-forming features have also been reported earlier [35]. Three different RHE types with balanced nucleotide composition were detected with our search criteria, one of them comprises 30 copies in different fragments sizes of up to 40 bp (Figure 4). The *Suberites domuncula *RHE are present inside *rns*, *rnl *and *nad5 *and in the IGRs 5' to *trnI(cau)*, *trnK*, *atp6*, *trnR(ucu), cox3, trnQ(uug), trnN(guu), cob, trnT(ugu), trnE(uuc), trnD(guc), trnR(ugc), trnH(gug), cox1, trnS(uga), nad1, trnL(uaa), nad2, nad5, trnA(ugc), trnF(gaa) *and *trnV(uac)*.

### Repetitive and other unusual elements in mtDNA of other poriferan lineages

Recent phylogenomic analyses suggested the presence of four major extant sponge lineages: Demospongiae, Calcarea, Hexactinellida and Homoscleromorpha [29].

#### Hexactinellida

The mt DNA of *Iphiteon panicea *and *Sympagella nux *are not completely sequenced due to the difficulties associated with PCR amplification of a single large non-coding region present in these genomes (see [36]), and hence may contain a number of undetected RHE. Therefore the Hexactinellida results have to be regarded with caution. The sequenced region of *Iphiteon panicea *(Hexactinosida, 1,551 bp IGR) does not possess any hairpin-forming repeat structures. Instead there are six repetitive regions of about 30 bp, which make up in some cases almost an entire IGR and overlap by 5 bp with the 3' regions of *nad3, nad4L *and *nad4*. A full-length copy of such repeat is inserted within *cob*. This 3' region of *cob *is highly variable and therefore potentially not relevant for the function of the gene. Other copies of the motif overlap with *trnI(gau) *and reside within *orf909*. Furthermore, several additional repeat units are present in *Iphiteon panicea*, which share up to 43 bp and a 7 bp core sequence. The complete mtDNA sequence of *Aphrocallistes vastus *(1,444 bp IGR, including one *orf*), another representative of the order Hexactinosida, does not possess RHE either. A large non-coding region has been proposed to be a control region [11]. This region contains several repetitive elements including a single (not repetitive) 28 bp hairpin motif consisting of two perfectly complementary 14 bp stretches, one 21 bp complementary repeat and a 21 bp region shared with the IGR upstream of *cox2*. Other repeated regions include three identical (non RHE-) elements of 41 bp in *rns*, *rnl *and the 5' terminus of *cox2*. The mt genome of *Sympagella nux *(Lyssacinosida, 1.011 bp IGR) does not possess RHEs in the sequenced portion, but contains a 117 bp repeated region including *trnD*, of which a copy is present 423 bp upstream between *atp9 *and *trnM(cau)*.

#### Homoscleromorpha

The mt genomes of the homoscleromorph *Plakortis angulospiculatus *(601 bp IGR) and *Oscarella carmela *(1,275 bp IGR) lack RHE according to our search criteria.

#### Calcarea

Yet, we lack any comprehensive information on calcarean (calcareous sponges) mt genomes.

## Discussion

### Major morphological differences are not reflected in molecular distances

**Figure 5:**
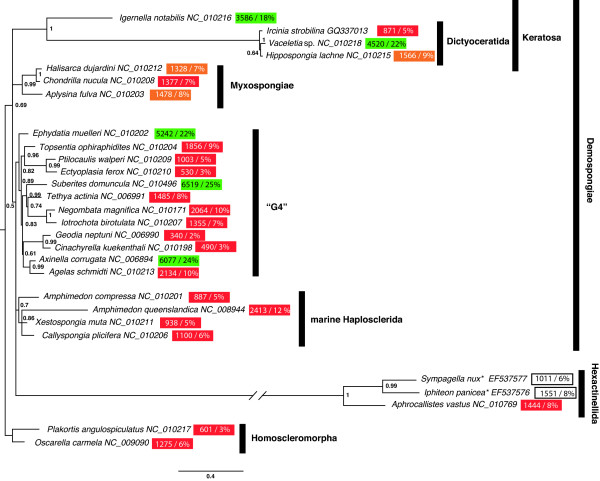
**Phylogenetic tree reconstructed from the protein coding genes of the mt genomes analysed**. Phylogenetic tree reconstructed from the protein coding genes. The coloured boxes following the taxon names comprise the numbers and ratios of nucleotides in the IGRs. Green boxes: presence of significant RHEs; orange boxes; presence of repetitive elements in very low copy numbers; red boxes: no RHEs; uncoloured boxes: undecided due to incomplete sequences. The numbers following the taxon names indicate the GenBank accession numbers. Numbers on the branches are Bayesian posterior probabilities. The asterisks at hexactinellid taxa denote incompletely sequenced genomes. Please note that the long branch of Hexactinellida might influence branching pattern and higher support values (refer to [5,29,30]).

It is remarkable that the mt genomes from three dictyoceratid species possess an identical gene content and gene arrangement and contain several similar IGR regions, because they represent taxa with very different skeletal types. *Vaceletia*, a taxon with sphinctozoan-like (chambered) bauplan features a massive, "hypercalcified" skeleton. Sphinctozoan-type sponges were metazoans that built large reef structures in the Palaeozoic and Mesozoic, together with other coralline sponges that built massive calcified skeletons with stromatoporoid, chaetetid, and pharetronid grade of construction (e.g. [37]). *Vaceletia *had been placed as the only extant genus in the order Verticillitida [38] and only molecular data revealed a dictyoceratid relationship of *Vaceletia *[5,31]. Among extant organisms, *Vaceletia *is the only animal that possesses this ancient sphinctozoan-type skeleton. Our data reveals that significant morphological difference of the sphinctozoan coralline sponge *Vaceletia *(with mineral skeleton), compared to the non-sphinctozoan dictyoceratids such as *Hippospongia *and *Ircinia *(with an organic skeleton) is not reflected in either nuclear ribosomal RNA genes [31] or in mt data (Figure 5). This is particularly interesting for understanding molecular phylogenies comprising several other extant coralline demosponges, which likewise construct a 'hypercalcified' secondary limestone skeleton in addition to their primary (often spicular) skeleton [39]. In the past, these coralline sponges had been lumped into a separate sponge class "Sclerospongiae" [40], but were subsequently assigned to several demosponge and calcarean orders, and are clearly polyphyletic [39].

The non-coralline Dictyoceratida *Hippospongia *and *Ircinia *share a considerable similarity in IGRs, which may suggest a more recent split between the two taxa. However, these species belong to two different families: Spongiidae and Irciniidae, each with a very distinct morphology: In contrast to Spongiidae, Irciniidae have very fine collagenous filaments with beaded ends in the mesohyl, which supplement the fibre skeleton and are unique among demosponges [41]. Such a well-defined autapomorphy is extremely rare among dictyoceratid sponges [42]. The genetic distances of mt genes do not indicate a particularly long evolutionary time for Irciniidae to develop this elaborated morphological feature.

The discrepancy between short branch lengths in phylogenetic trees inferred from mt data and a large extent of morphological divergence can have several explanations. On the one hand, it is possible that the morphological changes have taken place in a relatively short time span. This hypothesis is supported by previous findings on *Merlia normani*, (Poecilosclerida: Merliidae), a demosponge species known to possesses forms with and without a calcified basal skeleton [43]. The two forms of *Merlia normani *provided important evidence for understanding that calcified skeletons are frequently homoplasious and therefore weak characters for demosponge phylogenies [43]. Alternatively, the evolutionary rates in Dictyoceratida might have decreased dramatically since their radiation (see Figure 5). Additional keratose sponge mt data are needed to decide between these two possibilities.

### Reduction of the mitochondrial tRNA content in keratose sponges

The Dictyoceratida and their dendroceratid sister taxon *Igernella notabilis *retained only two tRNA genes in mtDNA necessary to compensate for the derivation from the universal genetic code: *trnW(uca) *in the sponge (as well as all other animal and many other eukaryote) mitochondria translates the opal codon UGA that specifies a termination signal in the standard genetic code [32]; *trnM(cau) *is a specialized initiation of translation (see also [33]). Cnidarian mt genomes likewise possess an identical set of tRNA genes. However, reduction in the cnidarian and dictyoceratid mt tRNA gene sets must have evolved independently given that both Myxospongia, the sister group of Keratosa, and Hexactinellida, the putative sister group of Demospongiae, possess a full complement of mt tRNA genes.

### Hairpin elements and the evolution of metazoan mt genomes

Our study demonstrates that repetitive inverted repeats with potential to form secondary structures such as hairpins, double hairpins or even more elaborate structures, are found repeatedly in demosponge mt genomes. Their presence in sponge mt DNA is remarkable because repetitive elements other than in the control region are hardly known from Bilateria (see also [44]). This observation underlines the intermediate appearance of sponge mtDNA [10,45] relative to Eumetazoa, in which such elements are almost unknown, and to non-metazoan opisthokonts such as fungi, in which repetitive elements are abundant.

A correlation between IGR length and the presence of repetitive elements is obvious (Figure 5): RHEs are more common in sponge taxa with an increased IGR length. However, an attempt to explain this correlation leads to the Aristotle's 'Chicken or the Egg?' question: Long IGRs present in most poriferan taxa provide more targets for the accumulation and fixation of RHE, in contrast to the greatly reduced IGRs in Eumetazoa. Insertion of RHE in reduced and highly economized mt genomes of bilaterian animals could almost exclusively take place in the coding regions only (besides the control region) and would very likely interfere with the functionality of crucial genes. As a consequence, a RHE will less likely be fixed in the population. Vice versa, the accumulation of repetitive elements causes a prolongation of IGRs, which reach up to 25% of the total genome size in demosponges. Nevertheless, we found many poriferan taxa without significant RHEs in their mt genomes, but with IGRs of considerable length (e.g. *Agelas schmidti*, *Amphimedon queenslandica*, *Chondrilla nucula*, *Negombata magnifica *and *Iotrochota birotulata*), which suggest that RHE are not the sole responsible elements for IGR length.

In a few cases RHEs are present in coding regions of the mt genomes - mostly in ribosomal RNA genes such as *rnl *(Additional file 1) and *rns *(Additional file 2), and less frequently in protein genes. Most insertions into ribosomal RNA genes have taken place at sites, in which extensive length differences have been reported earlier, and their presence may not have a significant influence on the function of the ribosomal RNA (see [46]). Therefore, excision of the elements out of the transcript may not be necessary to maintain the function of the RNA in the ribosomes. Studies with fungal mt double hairpin elements inserted in genes revealed that RHEs are not removed from the transcripts, which is consistent with their absence from structurally important portions of genes [12]. Likewise GC-rich clusters in the *var1 *ORF or in rRNAs of yeast mitochondria are neither removed nor edited at the RNA level [47-49].

### Taxonomic distribution of repetitive hairpin-forming elements in Porifera

Our analyses leave the question of whether the distribution of RHEs in poriferan mt genomes has any taxonomic preferences unresolved. It seems possible that some clades are more susceptible to invasions of RHEs than others - but additional poriferan mt genomes will be necessary to test this hypothesis. So far, Keratosa (c.f. [30]) and, to lesser extend, Myxospongiae (c.f. [30]) display a higher abundance of RHE compared to the clades of marine Haplosclerida and the 'G4' group (c.f. [30]). In the two latter, repetitive hairpin forming elements are only present in *Ephydatia muelleri, Axinella corrugata *and *Suberites domuncula*. For Hexactinellida, no unambiguous prediction is possible as only three taxa of two orders were investigated and two out of the three sequences are incomplete. Homoscleromorpha, which is a species-poor taxon [50], do not possess relevant repetitive structural elements, which therefore might not occur in this group. However, one of the two species of homosclermomorphs contains two introns - another type of "selfish" DNA - in *cox1 *[51].

Repetitive hairpin elements are not uncommon among demosponges and therefore putatively some could have been present in (now extinct) taxa diverging earlier from the lineage leading to the last common ancestor of Porifera. Furthermore, as ancestral mt genomes have likely had larger IGRs, they provided more target sites for insertions of RHEs within a mt genome, it is also possible that RHEs were present in (now extinct) taxa diverging earlier from the lineage towards the last common ancestor of Metazoa. Subsequent genome compression in the lineage towards Bilateria after the split of Porifera combined with the loss of IGRs prevented the infestations of RHE in higher metazoan mt genomes.

The scattered distribution of RHEs that we observed in the present study could either suggest an early origin with subsequent parallel loss, or multiple independent invasions. The latter possibility appears more plausible given the structural differences between RHE elements found among demosponge taxa. Consequently, repetitive hairpin-forming elements may have invaded metazoan mt genomes repeatedly during their evolution. They may be secondarily lost again in some poriferan lineages, but are, with the exception of the control region, mostly absent in eumetazoan mt genomes due to their compact organization with subsequent loss of the preferred target sites within the IGRs (but see also [44]).

### Evolution of repetitive hairpin-forming elements in Porifera

Large differences in secondary structures and nucleotide composition observed in RHEs of sponges suggest their independent origin and evolution. RHEs in the keratose sponges *Vaceletia*, *Hippospongia *and *Igernella *have an extremely high AT content. Both "*yellow18*" and "*blue15*" motifs in *Vaceletia *mtDNA likely have a common origin. It is possible that the short fraction of the "*yellow18*" type RHE evolved into the "*blue15*" type RHE, of which subsequently several copies independently inserted into other parts of the *Vaceletia *mt genome. Apparent double hairpins (which are known from other genomes) are likewise formed by tandem insertion of "*blue15*" and/or "*yellow18*" RHEs into the mt genome.

The stem regions of both, "*blue15*" and "*yellow18*" RHE types are conserved, while the loop regions display a few differences. We interpret this as an indication of either their recent origin and rapid spread through the genome or considerable pressure for maintaining a hairpin secondary structure and note that this pattern is in contrast with other structured RNAs, such as group I and group II introns [52,53], in which the loops tend to be more conserved than the helical regions [12]. This lack of sequence conservation in the loops in demosponge hairpin elements suggests their lack of structural importance and their unlikely involvement in any tertiary interactions [12]. This observation is supported by the substitution pattern in large repetitive, triple hairpin forming regions of *Igernella notabilis*, in which helix regions are also conserved and substitutions only occur in the loop regions.

The RHE found in other demosponges have a higher GC-content. In particular, the stem regions of RHE in *Halisarca dujardini *are up to 100% G+C. Substitutions only occur at the loop positions, which parallels to the structural constraints observed in the keratose RHE.

The lack of similarity between the RHE of different sponge taxa implies that they infested the mt genome in multiple, independent events rather than in a single infestation followed by proliferation into different elements. This inference is supported by the abundance of structural different repetitive hairpin-forming elements in fungi and other non-metazoan opisthokonts. In particular, the distinction into GC-rich and AT-rich elements raises evidence for at least two, but probably more infestation events in Porifera. This is consistent with many earlier observations that well-distinguishable structure forming repetitive elements are frequently confined to groups of closely related species, where the distribution indicates direct exchange of genetic material (see [12] for examples, but also for evidence for mobility).

The relatively conserved structures of sponge RHEs within the individual mt genomes suggest their recent multiplication and dispersal throughout the mt genome. However, we also have to consider that alternatively, reduced substitution rates in diploblast mt genomes, which are up to 10-20 times lower than their bilaterian counterparts [54] may contribute to the low number of base substitutions observed between copies of each element.

### Proliferation of repetitive hairpin-forming elements within poriferan mt genomes

The extensive repetitive and secondary-structure-forming regions in the *Vaceletia *mt genome provide insight into the intra-genomic dispersal of the hairpin elements. The large identical IRG clusters indicate that the hairpin elements are not necessarily only copied as single elements. Instead, larger motifs such as the 399 bp repetitive region ("*red399 motif"*, Figure 1) are likewise duplicated and inserted at different positions of the mt genome. Shorter fragments with high sequence identity to those fragments such as the 236 and 219 bp ("*red motifs*") fragment might be derived from a copy of the 339 bp counterpart and subsequently reduced after insertion as full length elements. The spread of RHEs in *Vaceletia *was apparently a rapid process compared to (and probably largely independent from) other genomic changes like substitutions and rearrangements in the gene order as evident by comparing the mt genomes of *Vaceletia *and *Ircinia*. The latter genome has an identical gene arrangement, but completely lacks RHEs.

Evidence for lateral transfer and inter-genomic mobility of repetitive hairpin elements could not be found due to the lack of sufficient population samples. In non-metazoan taxa inter-genomic mobility of RHE was hypothesized e.g., for fungi of the genus *Allomyces*, where closely related species possess different frequencies of RHE insertions [12] in congruence to previous observations in rice [16] and yeast [13,14]. Mechanisms for mobility of RHE may be different. Mobility of the yeast RHE, located in GC-rich clusters, is believed to happen by means of transposition at the DNA level similar to DNA transposons [14]. By contrast, a different mode of transposition, potentially via RNA intermediates, has been suggested for the *Allomyces *RHEs because of the lack of duplications in the flanking regions typical for DNA-transpositions [12]. For Porifera, the lack of mt sequences of closely related species yet prevents speculation on their RHE transposition mechanisms.

## Conclusion

Several poriferan mt genomes possess large IGRs, which are target sites for repetitive hairpin elements. RHEs themselves also contribute to the large size variation found among sponge mt genomes. Their scattered distribution and dissimilar structure strongly suggests multiple independent invasions of RHEs instead of a single ancestral event with subsequent loss in some lineages. Additionally, the presence of RHE- clusters in *Vaceletia *sp. implies a rapid proliferation in combination with intra-genomic mobility of such motifs.

As RHEs are not uncommon among extant demosponges, occasional RHE invasions might also have occurred in (now extinct) taxa diverging earlier from the lineage leading to the last common ancestor of Porifera. Furthermore, as ancestral mt genomes were probably richer in IGRs, and therefore provided more target sites for insertion of RHEs, it is likely that occasional RHEs infestations already occurred very early in metazoan mt genome evolution (and affected now extinct lineages). Subsequent genome compression in the lineage towards Bilateria after the split of Porifera combined with the loss of IGRs lead to the loss of RHE in eumetazoan mtDNA.

The mt genomes of Dictyoceratida provide information on metazoan mt genome evolution. The high nucleotide and structural similarity of the dictyoceratid mt genomes is opposed to the different morphology of its taxa, which must be accounted for in evolutionary studies on other poriferan groups with a similar degree of morphological differences.

## Methods

*Ircinia strobilina *was collected by Robert Thacker at the Smithsonian Tropical Research Institute's Bocas del Toro Research Station in Panama. Total DNA of *Ircinia strobilina *was extracted from ~0.2 g of tissue with a phenol- chloroform method modified from [55]. Porifera-optimized conserved primers for *cox1 *and *cox2 *[5] were used to amplify short fragments of these genes. Two species-specific primers were designed for each gene (is-cox1-f1, 5'-GGGAATAAGTTGAACTCGACTGC-3', is-cox1-r1, 5'-TACCGATAGACACCATGGCATAC-3', is-cox2-f1, 5'-AGAGGTGGACAACAGACTATTGC-3', and is-cox2-r1, 5'-TGATTTAATCTCCCTGGCACTGC-3') and complete mtDNA was amplified in two fragments ~6 and 10.5 kbp in size using the Long and Accurate (LA) PCR kit from TAKARA. The PCR amplifications were combined in equimolar concentration, sheared into pieces 1-2 kb in size and cloned using the TOPO^® ^Shotgun Subcloning Kit from Invitrogen. Colonies containing inserts were collected, grown overnight in 96-well blocks and submitted to the DNA Sequencing and Synthesis Facility of the ISU Office of Biotechnology for high-throughput plasmid preparation and sequencing. The STADEN program suite [56,57] was used to basecall and to assemble the sequences. Gaps in the assembly were filled by primer-walking using original PCR amplifications as templates. The repeats observed were too short to interfere negatively with the assembly process; see [58] also for other details of the shotgun plastid sequencing procedure. tRNA genes were identified with the tRNAscan-SE program [59]; other genes were identified by similarity searches in GenBank at NCBI using the BLAST network service [60]. The sequence of *Ircinia strobilina *is deposited to Genbank under accession number GQ337013.

For the phylogenetic reconstructions protein data of sponge mtDNA was aligned following previously published methods e.g. [5]: amino acid sequences of individual proteins (except *atp8*) were aligned three times with ClustalW 1.82 [61] using different combinations of opening/extension gap penalties: 10/0.2 (default), 12/4 and 5/1. The three alignments were compared using SOAP [62], and only positions that were identical among them were included in phylogenetic analyses. The final alignment comprised 3,576 amino acids. The phylogenetic tree of *Ircinia strobilina *and other complete mt genomes of Porifera (GenBank accession numbers have been incorporated into Figure 5) has been reconstructed with PHYLOBAYES 2.3 under the CAT + Γ model [63] with 4 chains and every 100th tree sampled after a burn-in of 1000. More than 9000 trees where sampled from each chain and the largest (maxdiff) and mean (meandiff) discrepancy observed across all bipartitions were maxdiff: 0.103701, meandiff: 0.00459985, which constitutes a good run according to the PHYLOBAYES manual. The rates of synonymous/nonsynonymous codon substitution rates were estimated with PAML 4.1 [64].

Artemis 9.0 [65] was used for genome visualization and handling, Codoncode Aligner v.2.0.6 http://www.codoncode.com for alignment. Repetitive features have been screened using PILER v.1 in combination with PALS v.1 [66]. In order to minimize false positives, but to perform sufficient thorough analyses we screened for motifs of at least 13 bp lengths with 95% identity. Positive hits were compared against GenBank with blastN [67] in order to find evidence for functionality or relationship to other published DNA fragments. RHE secondary structures were initially inferred under minimum free energy predictions from the mfold-server http://frontend.bioinfo.rpi.edu/applications/mfold/cgi-bin/rna-form1.cgi.

## List of abbreviations

bp: basepair(s); IGR: intergenic regions; mt: mitochondrial; RHE: repetitive hairpin-forming element.

## Authors' contributions

DE designed the study, analysed the data and drafted the manuscript. OV participated in the analysis, in particular of the secondary structures and critical revision of the manuscript. GW contributed to the conception and design of the study, and critical revision of the manuscript. DVL determined mtDNA sequence of *Ircinia strobilina*, provided RHE data for *Axinella corrugata*, conducted phylogenetic analysis, and contributed to the writing. All authors read and approved the final manuscript.

## Supplementary Material

Additional file 1 **16S of *Axinella corrugata***. Secondary structure prediction of the mt *rnl *(16S ribosomal RNA gene) of *Axinella corrugata *as modified from [51]. RHEs are labelled in red. Regions prone to large length differences in *rnl *of other eukaryote taxa (cf. [46]) are shaded in grey. Click here for file

Additional file 2 **12S of *Vaceletia *sp**. Secondary structure prediction of the mt *rns *(12S ribosomal RNA gene) of *Vaceletia *sp. RHEs are labelled in red. Regions prone to large length differences in *rns *of other eukaryote taxa (cf. [46]) are shaded in grey. Click here for file
